# Human papilloma virus vaccination in the resource-limited settings of sub-Saharan Africa: Challenges and recommendations

**DOI:** 10.1016/j.jvacx.2024.100549

**Published:** 2024-08-20

**Authors:** Grant Murewanhema, Enos Moyo, Mathias Dzobo, Rachel S. Mandishora-Dube, Tafadzwa Dzinamarira

**Affiliations:** a Unit of Obstetrics and Gynaecology, Faculty of Medicine and Health Sciences, University of Zimbabwe, Zimbabwe; b School of Nursing and Public Health, College of Health Sciences, University of KwaZulu-Natal, South Africa; c School of Health Systems and Public Health, University of Pretoria, South Africa; d Department of Laboratory Diagnostics and Investigative Sciences, Medical Microbiology Unit, Faculty of Medicine and Health Sciences, University of Zimbabwe, Zimbabwe

## Abstract

Human papillomaviruses (HPV) cause 99% of all cervical cancer cases globally, with the high-risk genotypes 16 and 18 causing at least 70% of these cases. An estimated 90% of the global cervical cancer burden occurs in low-to-middle-income countries (LMICs), particularly in sub-Saharan Africa (SSA). Primary prevention through the administration of efficacious HPV vaccines is key to the World Health Organization’s global strategy for accelerating the elimination of cervical cancer as a disease of public health concern. The rollout of HPV vaccination in SSA is faced with several challenges, such as the high cost of vaccine procurement, a lack of funding and political will from the central governments of countries, and inadequate infrastructure for vaccine cold chain storage and transport. Stigma, misinformation, lack of education and awareness, and vaccine hesitancy constitute the social factors that affect the successful rollout or implementation of vaccination programs in SSA. Based on the challenges SSA faces in rolling out HPV vaccination, we recommend using strategies that address both the demand-side and supply-side obstacles to HPV vaccination uptake. These include costs and availability, fighting vaccine hesitancy, and increasing vaccine confidence.

## Introduction

Cervical cancer remains one of the cancers with marked global health significance. The World Health Organization (WHO) estimates that 604,000 women were diagnosed with cervical cancer in 2020, with an estimated 342,000 women dying from the cancer [1]. Low-to-middle-income countries (LMICs) carry the greatest burden of cervical cancer, accounting for 90 % of the new cases and deaths [1]. Sub-Saharan Africa (SSA), with its myriad of public health challenges, bears the brunt of the burden [2], and in several countries in this region, cervical cancer is the leading gynecological malignancy.

The etiology of greater than 99 % of invasive cervical cancers has been unequivocally established as persistent infection with high-risk human papillomavirus (HR-HPV) genotypes [3]. The HR-HPV genotypes 16 and 18 account for at least 70 % of all cervical cancers globally, whilst the other genotypes such as 31, 33, 35, 45, 56, and 58 account for the remaining burden [3]. Persistent infection is the risk factor for oncogenesis, with changes leading to invasive cancer occurring over 15–20 years in immunocompetent women. However, untreated HIV infection reduces the time it takes for HPV infection to develop into invasive cancer to 5–10 years [1].

The Global Cancer Observatory (GLOBOCAN) estimates show the disproportionate burden of cervical cancer across the different economic regions of the world as well as the differences in relative mortality. The current age-standardized incidence rates of cervical cancer in some LMICs in SSA are as high as 25 cases per 100,000 women to as high as 61.7 cases per 100,000 women in some countries, such as Zimbabwe [4]. An estimate of the five-year age-standardized relative survival of cervical cancer shows a 33 % survival in LMICs, in sharp contrast with the 67 % observed in high-income countries [5].

The WHO 90–70-90 strategy envisions the elimination of cervical cancer as a disease of public health concern, with a reduction of the age-standardized incidence rate to less than 4 per 100,000 women globally, from age-standardized rates of between 75 per 100,000 women in the highest-risk countries and fewer than 10 per 100,000 women in the lowest-risk countries [6]. The 90–70-90 targets mean that 90 % of girls should be fully vaccinated with an effective HPV vaccine by the age of 15 years, 70 % of women should be screened with a high-performance test by the age of 35 years, and again by the age of 45 years, and 90 % of women identified with cervical cancer should receive treatment [6].

Primary prevention of cervical cancer through vaccination for human papillomavirus (HPV) and secondary prevention through screening is critical for reducing the burden of cervical cancer in LMICs. Optimizing HPV vaccination programs in resource-limited settings is important so that maximum benefits, such as cervical cancer elimination, can be realized. In this article, we discuss the landscape of HPV vaccination in resource-limited settings, with a focus on SSA. We discuss the barriers to HPV vaccination, lessons learned from countries in SSA that have implemented national HPV vaccination programs, and the future of HPV vaccination. We base our discussion on a narrative review of the different available sources of literature at our disposal.

### Cervical cancer in sub-Saharan Africa

The world's highest rate of cervical cancer is in SSA [7]. Among the 20 countries in the world with the highest burden of cervical cancer, 19 are in the SSA [8]. The high rate of HIV infection in the SSA exacerbates the burden of cervical cancer [9]. Approximately 63 % of the 39 million people living with HIV (PLHIV) worldwide in 2022 were from SSA. SSA was responsible for 440,000 of the 1.2 million new infections [10]. Compared to women without HIV, women living with HIV (WLHIV), including those on treatment with highly active antiretroviral therapy, are six times more likely to develop cervical cancer [4]. The dual challenge of living with HIV and the risk of developing cervical cancer places significant financial resource constraints on countries in SSA. The costs of yearly screening for women living with HIV are considerably higher compared to HIV-negative women, who require less frequent screening [11]. The high burden of cervical cancer in SSA is also attributed to a lack of access to national screening programs due to financial and logistic challenges [12]. Countries like Malawi, Zambia, eSwatini, Tanzania, Zimbabwe, and Lesotho have age standardised incidence rates (ASIR) that exceed 40 per 100,000 [7]. In 21 of the 48 SSA countries, cervical cancer is the leading cause of cancer-related deaths among females [7]. Table 1 below provides a summary of the burden of cervical cancer in countries in the SSA.

**Table 1:** Cervical cancer burden in sub-Saharan Africa.

**Country**	**Cervical cancer age-standardized incidence rate**^a^ **(per 100,000 women per year)**	**National HPV vaccination program available**^b^
Angola	37.6	No
Benin	15.1	No
Botswana	34.4	Yes
Burkina Faso	18.2	Yes
Burundi	49.3	No
Cape Verde	17	Yes
Cameroon	**33.7**	Yes
Central African Republic	21.8	No
Chad	20.2	No
Comoros	56	No
Congo	22.4	No
Cote d’Ivoire	31.2	Yes
Democratic Republic of Congo	31.9	No
Djibouti	15.3	No
Equatorial Guinea	32.9	No
Eritrea	15.3	Yes
Eswatini	84.5	Yes
Ethiopia	21.5	Yes
Gabon	30.8	No
Ghana	27.4	No
Guinea	50.1	No
Guinea-Bissau	39.6	No
Kenya	31.3	Yes
Lesotho	56.8	Yes
Liberia	40.8	Yes
Madagascar	41.2	No
Malawi	67.9	Yes
Mali	36.4	No
Mauritania	28.9	Yes
Mauritius	12.6	Yes
Mozambique	50.2	Yes
Namibia	37.4	No
Niger	10.4	No
Nigeria	34.6	Yes
Rwanda	28.2	Yes
Sao Tome and Principe	16	Yes
Senegal	36.3	Yes
Sierra Leone	21.2	Yes
Somalia	25.1	No
South Africa	35.3	Yes
Sudan	8.7	No
Tanzania	62.5	Yes
The Gambia	42.9	Yes
Togo	19.1	Yes
Uganda	56.2	Yes
Zambia	65.5	Yes
Zimbabwe	61.7	Yes

a Data obtained from: International Agency for Research on Cancer [13].

b Data obtained from: [14] and [15].

### Human papilloma virus vaccines

There are currently six licensed prophylactic HPV vaccines available globally, all produced using recombinant DNA technology. Table 2 below provides a summary of these.

**Table 2:** Available HPV vaccines.

**Vaccine brand name**	**Manufacturer**	**Type of vaccine**	**HPV types covered**
Cervarix	GlaxoSmithKline	Bivalent	16 and 18
Cecolin	Xiamen Innovax Biotech	Bivalent	16 and 18
Walrinvax	Zerun Biotech	Bivalent	16 and 18
Gardasil	Merck	Quadrivalent	6, 11, 16, and 18
Cervavac	Serum Institute of India	Quadrivalent	6, 11, 16, and 18
Gardasil-9	Merck	Nonavalent	6, 11, 16, 18, 31, 33, 45, 52, and 58

The three bivalent vaccines are Cervarix, made by GlaxoSmithKline, Cecolin, made by Xiamen Innovax Biotech, and Walrinvax made by Zerun Biotech. There are two quadrivalent vaccines, namely Gardasil produced by Merck, and Cervavac produced by the Serum Institute of India, and one nonavalent vaccine, Gardasil-9, made by Merck. These vaccines induce effective protection against HPV infection by triggering humoral immunity, producing virus-neutralizing antibodies, and preventing viruses from entering host cells [16]. They are all administered intramuscularly. Although the six vaccines are available internationally, only Cervarix, Gardasil, and Gardasil-9 are currently available in SSA.

Even one or two doses may provide lifetime protection to sexually naïve adolescent girls and boys, even though three dose regimes were previously advised [17]. The International Agency for Research on Cancer (IARC)’s recent single vaccine dose study using Gardasil provides strong evidence for the efficacy of one dose of the bivalent vaccine [18]. The Advisory Committee on Immunization Practice (ACIP) in 2016 recommended two doses for persons less than 15 years and three doses for the immunocompromised and those who are vaccinated after the age of 15 years [19]. The WHO now recommends a one-dose regimen as it is more cost-effective compared to a two-dose regimen [20]. Sixteen countries in Africa have already endorsed a single-dose regimen in their national HPV vaccination programs [15].

Several studies revealed that HPV vaccination using bivalent and quadrivalent vaccines is cost-effective, even in LMICs like those in SSA [21], [22]. According to one study, a one-dose HPV vaccine program is more cost-effective compared to a two-dose regimen as it simplifies vaccine delivery and reduces costs [23]. Another systematic review revealed that HPV vaccination may be cost-effective for LMICs if the total cost per vaccinated child is US$10 to US$25 [21]. A study on the nonavalent vaccine's cost-effectiveness in comparison to the bivalent and quadrivalent vaccines conducted in Kenya and Uganda revealed that the nonavalent vaccine is cost-effective [24]. However, GAVI, a company that provides vaccine subsidies for LMICs, is subsidizing Gardasil-9 in Kenya and Uganda, which may have improved its cost-effectiveness. Despite being cost-effective, global HPV vaccination coverage is still low. In 2018, it was estimated that the HPV vaccination global coverage was about 12 %. The coverage was higher in high-income countries (HICs) compared to LMICs [25].

### Barriers to HPV vaccination

**Fig. 1:**
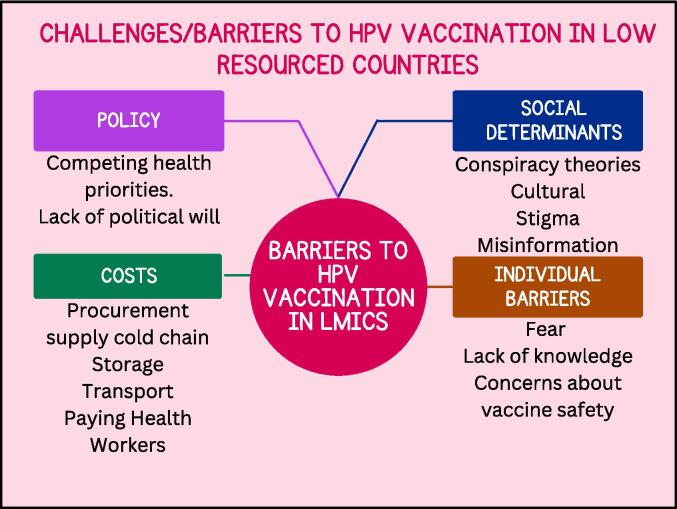
Challenges/barriers to HPV vaccination in SSA.

The rollout of HPV vaccination in LMICs is faced with several challenges, such as the high cost of vaccine procurement, a lack of funding and political will from the central governments of countries, and inadequate infrastructure for vaccine cold chain storage and transport [26], [27], [28]. Stigma, misinformation, lack of education and awareness, and vaccine hesitancy constitute the social factors that affect the successful rollout or implementation of vaccination programs [26]. The other challenges/barriers to HPV vaccination in LMICs are presented in Fig. 1.

For many developing countries, the procurement of HPV vaccines is costly, and in the absence of relevant global partnerships, including strategic funding and supply agreements to guarantee affordable prices and reliable vaccine supply, HPV vaccination will remain an unmet priority. In most developing countries, budgetary provisions for health needs are often thin due to various competing priorities. Additionally, there is a lack of political will and urgency to fund HPV vaccine procurement and delivery because cervical cancer manifests years later after infection [29]. It is possible that countries would rather give priority to funding programs for HIV and TB that have an immediate urgency [8].

The integration of HPV vaccination into national immunization programs poses a challenge in some developing countries due to the unique target population for HPV vaccine (9–14 years), which is different from the usual target for traditional vaccination programs, which target zero to five-year-old children [30]. The rollout of HPV vaccination has heavily used the school-based system that targets adolescent girls aged 9–14 years [27]. A study conducted in Tanzania revealed that vaccination coverage for the first dose in a school-based program was higher than 80 % [31], while another school-based program conducted in Uganda achieved coverage of more than 95 % [32]. The school-based program approach has reported successes in many developing countries, but it also suffers from challenges such as school dropout, natural disasters, wars, and instability, and it is a challenge to implement in places that have low attendance or enrolment of children in formal education [27].

The additional costs of maintaining a cold chain for the storage and transport of vaccines result in increased costs, which many developing countries are unable to fund. The maintenance of a cold chain is important to preserve the potency and effectiveness of the vaccine before administration [33]. Getting the vaccines to remote areas is problematic due to poor and dilapidated road infrastructure.

The need for the delivery of HPV vaccinations overburdens the outstretched healthcare workforce in developing countries. Existing health challenges mean there are insufficient health workers to attend to other needs, such as HPV vaccination programs. In the absence of adequate training, education, and financial incentives for health workers, the implementation of HPV vaccination faces slow progress in many developing countries [29].

Vaccine hesitancy in developing countries presents a serious barrier to HPV vaccination rollout. The WHO views vaccine hesitancy as one of the top 10 threats to global health [34]. Rumors, falsehoods, myths, misconceptions, and conspiracy theories that surrounded COVID-19 vaccination may have exacerbated the challenge of HPV vaccine hesitancy.

Despite evidence that points to the benefit of vaccinating sexually naïve adolescent girls, parental beliefs about the appropriateness of pre-adolescent immunization limit the uptake of HPV vaccines among school-going girls [30]. There are concerns by parents of losing autonomy over their children’s welfare and health matters since the HPV vaccination program at school is often compulsory. Another concern raised by parents is the fear that children will engage in early sexual activities, thinking that they have blanket protection from all sexually transmitted infections after HPV vaccination [35]. Fear over vaccine safety, due to misinformation, affects the uptake of the HPV vaccine in communities, e.g., the HPV vaccine has been reported to cause sterility in people who receive it [36].

### Lessons learned from SSA countries that have implemented national HPV immunization programs

Several lessons have been learned from countries that have already introduced HPV vaccination into their national immunization programs. A study conducted in six African countries that had introduced a national HPV vaccination program in 2016 noted that since the routine target for HPV vaccination of 9–14-year-old girls is not targeted for vaccination in SSA, it is possible to select girls for vaccination using their school grade as opposed to their specific ages [37]. The study also reported that schools can be successfully used as venues for HPV vaccination, provided primary school enrolment is high in the countries. Other lessons learned from this study were that political endorsement of the HPV vaccination program was critical to community acceptance and that integration with other school health programs reduced costs [37].

A study conducted in Malawi reported that there were concerns among caregivers about the effectiveness and side effects of HPV vaccines [38]. However, the use of schools as vaccination sites was noted to counteract fears and rumors associated with the HPV vaccines because schools are trusted environments. The school-based HPV vaccination program was also preferred because it lessened the financial burden of traveling to healthcare facilities [38].

Limited domestic funding initially made the introduction of HPV vaccination in Senegal difficult [39]. However, support from GAVI ensured the introduction of the vaccine nationally [39]. The success of the national HPV vaccination program was partly attributed to close engagement and collaboration among different stakeholders, such as the ministries of health and education, as well as international partners such as the WHO, the United Nations Population Fund (UNFPA), and the United Nations Children’s Fund (UNICEF), among others [39]. Advocacy, communication, and social mobilization ensured an increase in uptake of the vaccine, while the engagement of community and religious leaders reduced misinformation and vaccine hesitancy [39]. Furthermore, the use of the ‘opt-out’ approach in schools ensured that more eligible girls were vaccinated. Senegal used already existing cold chain and waste management infrastructure, and this meant that no additional changes were made when HPV vaccination was introduced nationally [39].

## Strategies to optimize HPV vaccination in LMICs in sub-Saharan Africa

Based on the challenges SSA faces in rolling out HPV vaccination, we recommend using strategies that address both the demand-side and supply-side obstacles to HPV vaccination uptake.

### Government policy and political will

Strategies to address supply-side barriers include advocating for a strong political commitment by governments in the region, as this might ensure adequate funding for the HPV vaccination programs. Governments may mobilize resources from diverse stakeholders, including the corporate sector and non-governmental organizations, as a result of a strong political commitment. Additionally, a strong political commitment may guarantee effective interministerial coordination between the ministries of health and education, allowing for a variety of vaccine delivery techniques, including programs centered in healthcare facilities and schools. Such an approach may increase the accessibility of vaccines for both school-going and out-of-school adolescent girls [40]. In addition, most countries in SSA are eligible to receive HPV vaccination program funding from GAVI, and, they should, therefore apply for such funding [41].

Governments in SSA should adopt the one-dose vaccination schedule because it has the potential to increase coverage and completion rates at a lower cost than the two- or three-dose regimens (“WHO,” 2023). Integrating HPV vaccination programs within pre-existing EPI programs is a possibility, and strengthening pre-existing vaccination programs is a government prerogative. Without the political will and commitment of central governments, HPV vaccination programs may not be as successful.

### Cost and logistics

Making sure that the vaccines are provided to adolescent girls for free or at a low cost can also lead to increased coverage and accessibility. In the private health sector, medical insurance providers should be encouraged to pay for the vaccines fully, without extra payment required [40]. Catch-up vaccination programs may also help reach those who would have missed the vaccines [40].

Countries in SSA have marked experience administering vaccines to children through the expanded program of immunization (EPI) which has led to significant reductions in the incidence and burden of vaccine-preventable infectious diseases. HPV vaccination programs must be leveraged based on the experiences and lessons learned from implementing EPI programs, established cold chain storage systems, and healthcare worker experiences. Some of the challenges that countries in SSA have experienced during EPI programs that need to be addressed include vaccine refusals due to myths about the vaccines, inadequate cold-chain equipment at the healthcare facilities, a lack of functioning vehicles for transporting the vaccines to healthcare facilities, and inadequate initial sensitization of the target population and their guardians or parents [42], [43]. Additionally, and especially deriving from lessons learned from the COVID-19 pandemic, such as limited funds to procure the vaccines, concerns around vaccine safety, and the inability to access vulnerable populations [44], countries in LMICs must work hard to develop the capacity and infrastructure for local production of HPV vaccines, which may substantially lower the cost of vaccines. Moreover, school-based programs should be embedded into existing healthcare facility programs to reduce cost and logistic challenges.

### Addressing the social determinants of uptake

Healthcare providers should be provided with information about the vaccines so that they can be confident in recommending them to adolescent girls and their families [45]. The just-ended COVID-19 pandemic provides strong evidence of how societal myths, misconceptions, rumors, and conspiracy theories can widely propagate vaccine hesitancy. Carrying out qualitative and implementation science research is important to contextualize the societal determinants of vaccine uptake. Frequent promotional campaigns should also be conducted. These strategies may increase stakeholders’ knowledge and awareness about the risks of HPV infection and the benefits of HPV vaccination. Stakeholders' increased knowledge and awareness may boost public confidence in vaccinations by clearing up common misconceptions about them [46].

The public should be informed about immunizations through digital and social media platforms, churches, and other religious organizations using influential people, community champions, and cervical cancer survivors, as well as fact sheets distributed in public areas like schools [8]. It is important to improve vaccine education and communication to raise the demand for HPV vaccines. Maintaining multi-level communication with all of the stakeholders may also improve demand generation. Individuals’ understanding of their risk of developing cervical cancer is a critical determinant of vaccination uptake, just like the uptake of other health promotion activities. Providing sexual and reproductive health education is an element that must be integral to education curricula globally, sensitizing young girls about their risk of cervical cancer and the need for vaccination. Once adolescent girls are provided with adequate information about the vaccines, they may demand the vaccines themselves [47]. Religious barriers need to be addressed adequately, with several studies showing that in SSA, some religious sects exhibit poor uptake of health promotion and protection interventions. Specific strategies tailor-made to address the challenges in this sector are important and must be given due consideration in designing programs, including HPV vaccination in SSA.

## Conclusion

The WHO’s global strategy for the elimination of cervical cancer as a disease of global health significance cannot be realized if HPV vaccination programs in LMICs in the SSA are not optimized. Reducing the rate of cervical cancer globally requires optimal uptake of primary prevention strategies like HPV vaccination and secondary prevention like screening and treatment of precancerous lesions. The rollout of HPV vaccination in SSA is faced with several challenges, such as the high cost of vaccine procurement, a lack of funding and political will from the central governments of the countries, and inadequate infrastructure for vaccine cold chain storage and transport. Stigma, misinformation, lack of education and awareness, and vaccine hesitancy constitute the social factors that affect the successful rollout or implementation of vaccination programs in the region. Hence, concerted efforts from the global health community are needed to ensure continued HPV vaccine uptake and availability in SSA and make sure they catch up with the developed world in reducing the burden of cancer.

## CRediT authorship contribution statement

**Grant Murewanhema:** Writing – original draft, Conceptualization. **Enos Moyo:** Writing – original draft. **Mathias Dzobo:** Writing – review & editing. **Rachel S. Mandishora-Dube:** Writing – review & editing. **Tafadzwa Dzinamarira:** Writing – original draft, Conceptualization.

## Declaration of competing interest

The authors declare that they have no known competing financial interests or personal relationships that could have appeared to influence the work reported in this paper.

## Data Availability

No data was used for the research described in the article.
